# Impact of Different COVID-19 Pandemic Phases on Emergency Department Length of Stay and Intensive Care Unit Admission Risk: Single-Center Retrospective Cohort Study

**DOI:** 10.2196/87597

**Published:** 2026-08-11

**Authors:** Zehua Wang, Xiangmei Zhao

**Affiliations:** 1 Emergency Department, Henan Provincial People's Hospital, People’s Hospital of Zhengzhou University, 7 Weiwu Road, Zhengzhou, Henan, 450000, China, 86 13838585396

**Keywords:** COVID-19 pandemic, emergency department, ED, length of stay, LOS, intensive care unit admission risk, ICU admission risk, retrospective cohort study

## Abstract

**Background:**

COVID-19 profoundly affected emergency department (ED) services and patient outcomes. However, changes in ED length of stay (LOS) and intensive care unit (ICU) admission risk before, during, and after the pandemic remain poorly understood.

**Objective:**

We investigated and compared ED LOS and ICU admission risk among patients in the ED resuscitation area in 2019, 2021, and 2023, respectively before, during, and after the pandemic.

**Methods:**

This single-center retrospective cohort study enrolled patients in the ED resuscitation area of a regional emergency center in central China. ED LOS was compared across pandemic phases using the Kruskal-Wallis *H* test with Bonferroni correction. Cox proportional hazards models were applied to evaluate the association between phases and ICU admission risk. Sensitivity analyses included 2021 stratification, exclusion of extreme ED LOS values, and E-value analysis to validate result robustness.

**Results:**

A total of 44,161 patients were included. Stratified analyses demonstrated significant temporal variations in ED LOS across disposition groups. Among discharged patients, median ED LOS increased from a prepandemic 248 (IQR 149-404.8) minutes to a midpandemic 262.0 (IQR 153-423.8) minutes (*P*=.047) and remained elevated in the postpandemic phase (median 258, IQR 152‐451 minutes; *P*<.001). Among patients admitted to general wards, median ED LOS nearly doubled during the pandemic (from 186, IQR 96-431 to 350, IQR 188-610 minutes) and partially decreased to a postpandemic 293 (IQR 113-840) minutes (all *P*<.001). Among ICU-admitted patients, median ED LOS increased from a prepandemic 95 (IQR 57-169) minutes to a midpandemic 171 (IQR 77-306) minutes and a postpandemic 105 (IQR 50-215) minutes (all *P*<.003). The overall ICU admission rate was 15.8% (6982/44,161). Multivariate Cox regression with 2019 as the reference showed that the postpandemic phase was independently associated with an increased ICU admission risk (hazard ratio [HR] 1.079, 95% CI 1.012-1.150; *P*=.02), whereas the pandemic phase was not (HR 1.032, 95% CI 0.967-1.100; *P*=.34). In female patients, the postpandemic phase was associated with a higher ICU admission risk (HR 1.143, 95% CI 1.027-1.273; *P*=.02). All sensitivity analyses verified the robustness of the main findings (all *P*<.05).

**Conclusions:**

The COVID-19 pandemic exerted differential effects on ED LOS across disposition groups. The postpandemic period was associated with an increased risk of ICU admission, especially among female patients, whereas no significant change was observed during the pandemic. As a novel gender-stratified analysis covering the full pandemic timeline, this study distinguishes itself from prior research by revealing the sustained impacts of pandemic control policies on emergency care. Our findings deliver key evidence for patient risk stratification and offer practical guidance to optimize health care resource allocation in the postpandemic era. Health care systems should strengthen early identification of high-risk ED patients and rationalize critical care resource deployment accordingly.

## Introduction

Since 2020, COVID-19 has spread rapidly worldwide, triggering a global pandemic and posing a substantial threat to public health [1]. In response to the outbreak, governmental authorities implemented a series of public health interventions, including large-scale polymerase chain reaction screening, the establishment of fever clinics and isolation wards, the promotion of telemedicine services, and the enforcement of traffic restrictions, social distancing, and home isolation policies [2]. Public health emergencies frequently alter individual health care–seeking behaviors and disease management patterns [3]. As a frontline component of the health care system, emergency medical services (EMS) and emergency department (ED) operations have undergone substantial restructuring during the COVID-19 pandemic, with notable shifts in patient demographics, risk of intensive care unit (ICU) admission, and clinical outcomes. Previous studies have demonstrated that the incidence of stroke presentations to EDs decreased significantly during the pandemic, accompanied by elevated mortality rates [4]. The pandemic has also been associated with a relative increase in EMS activations related to acute cardiac events [5]. In South Korea, the pandemic resulted in a marked reduction in the number of patients transferred to ICUs via EDs, along with increased in-hospital mortality [6]. Similarly, the rate of ED visits for ankle trauma decreased notably during the pandemic, whereas the admission rate of patients with severe trauma increased significantly [7]. Another study reported that the COVID-19 pandemic led to a significant increase in the incidence of out-of-hospital cardiac arrest (OHCA) and a corresponding decline in survival rates [8].

In China, the COVID-19 pandemic evolved through 3 distinct phases. Following confirmation of human-to-human transmission on January 20, 2020, the government implemented strict Class A infectious disease control measures, including citywide lockdowns and travel restrictions [9]. These interventions rapidly contained the initial outbreak by May 2020 but caused severe disruptions to health care services and a sharp decline in ED visits [10]. Calendar year 2021 represented a relatively stable period under China’s “dynamic zero-COVID” strategy, characterized by standardized nonpharmaceutical interventions (NPIs) without the chaotic disruptions of the early outbreak or the extreme pressure of the Omicron surge [11]. In January 2023, the government adjusted the COVID-19 pandemic management measures to “Class B management,” marking a new phase in epidemic prevention and control [12]. The evolving prevention and control measures across different phases of the COVID-19 pandemic exerted differential effects on ED management models, which may have led to substantial changes in ED length of stay (LOS), clinical disposition, risk of ICU admission, and long-term prognosis. In Xi’an, China, ED visits for facial injuries decreased by 17% during the postpandemic period compared with the prepandemic period, accompanied by a significant delay in the time from injury to hospital presentation [13]. A study from northern China focusing on patients with infective endocarditis (IE) reported a striking increase in in-hospital mortality from 7.7% in 2019 to 26.4% in 2023 [14]. Similarly, patients undergoing hemodialysis infected with the Omicron variant of SARS-CoV-2 exhibited an elevated mortality risk and prolonged hospital stays [15].

ICU resources play a critical role in the treatment of critical illness and in responding to public health emergencies [16]. ICU admission serves as a primary outcome measure for evaluating health care system responsiveness and resource allocation efficiency [17]. Multiple studies have confirmed gender disparities in patient outcomes during ICU care [18,19]. Yang et al [20] demonstrated that the biological and social determinants driving disease deterioration during the Omicron-predominant phase differ between the sexes. Nevertheless, current research has predominantly focused on changes in ED use patterns before and during the pandemic, with limited data available for the postpandemic phase after the lifting of public health restrictions. Moreover, most existing studies are limited by relatively small sample sizes and population-specific sociodemographic and cultural contexts, leading to restricted generalizability and interpretability.

Therefore, the objective of this study was to describe and compare the risk of ICU admission among patients with ED resuscitation area across 3 distinct periods: the prepandemic (2019), pandemic (2021), and postpandemic (2023) periods. Using a retrospective cohort design, we also assessed temporal changes in ED LOS and disposition patterns. Prespecified sex-stratified analyses were performed. No causal hypotheses were tested; the study was descriptive and hypothesis generating in nature.

## Methods

### Study Design

This was a retrospective observational cohort study that enrolled patients treated in the emergency resuscitation area across 3 predefined periods: the prepandemic, COVID-19 pandemic, and postpandemic periods. The study design was observational with no experimental intervention, and all data were derived from routine clinical electronic medical records (EMRs).

### Study Setting and Population

The study was performed at a comprehensive emergency center serving a densely populated region in central China, covering approximately 90 million people within a 300-km radius. The annual ED volume is approximately 100,000 cases, with about 15,000 annual visits to the emergency resuscitation area. Patients were triaged by experienced nurses using a validated 5-level triage system. Only patients triaged as level I or II and admitted to the emergency resuscitation area were included [21]. During the pandemic period, all confirmed COVID-19 cases were transferred to designated hospitals and were not treated at the study ED.

We therefore selected 2021 as the representative pandemic period to reflect the typical impact of the pandemic under a consistent and sustained containment policy. This design provides a clear baseline for comparison with the prepandemic (2019) and postpandemic (2023) periods, when the country officially shifted to “Class B management” in January 2023 [12].

### Inclusion and Exclusion Criteria

Patients were included if they had received treatment in the emergency resuscitation area; had triage level 1 or 2; admission between January 1 and December 31 in 2019, 2021, or 2023; and available data for the primary outcome and key covariates.

Patients were excluded if there was a missing primary outcome (ICU admission), a complete absence of key demographic or laboratory covariates, or records with logical inconsistencies or biologically implausible values.

### Participant Characteristics

A total of 44,161 patients were included in the final analysis after the exclusion of 767 invalid records. Participant characteristics included age (continuous, years), sex (male or female), ED LOS, vital signs, laboratory results, and clinical disposition. Baseline characteristics were compared across the 3 study periods.

### Sampling Procedures and Data Collection

Participants were consecutively enrolled during 3 calendar years representing distinct public health phases: prepandemic phase (January 1, 2019, to December 31, 2019), COVID-19 pandemic phase (January 1, 2021, to December 31, 2021; stable dynamic zero-COVID phase), and postpandemic phase (January 1, 2023, to December 31, 2023; after implementation of Class B management). Data collection was retrospective, based on routine clinical care, with no participant self-selection bias. No additional interventions, examinations, or payments were provided to participants for research purposes.

### Sample Size and Data Diagnostics

The final analytic sample included 44,161 patients (2019: n=13,351, 30.23%; 2021: n=14,528, 32.9%; and 2023: n=16,282, 36.87%). No formal sample size calculation was performed because this was a retrospective real-world study using all eligible patients. Patients with missing primary outcomes or completely missing key covariates were excluded. Only 38 (0.09%) cases had missing ED disposition; complete case analysis (CCA) was used due to the negligible proportion of missing data. As a sensitivity analysis, multiple imputation by chained equations (MICE) was performed, generating 20 imputed datasets under the assumption of missing at random (MAR). The imputation model included all analysis variables (outcome, exposure, covariates, and censoring indicator). Pooled results were derived using the Rubin rules. The results of the CCA and the imputed analysis were consistent, as shown in Table S1 in Multimedia Appendix 1. Statistical outliers and biologically implausible values were identified and excluded before analysis.

### Measures and Covariates

The primary outcome was ICU admission, defined as transfer to an ICU for critical care management after initial treatment in the ED resuscitation area. ICU admission was treated as a binary time-to-event outcome in the Cox proportional hazards analysis.

ED LOS was defined as the interval from arrival at the resuscitation area to a predefined end point. For the primary time-to-event analysis (ICU admission), the end point was the first documentation of ICU admission; censoring events included ED discharge, transfer to a general ward, non-ICU death, or completion of the study period. For the secondary descriptive analysis of ED LOS, the end point was physical departure from the ED, irrespective of final disposition (discharge, general ward admission, ICU admission, or other outcomes).

Covariates were selected based on existing literature and clinical relevance. The following prespecified variables were forced into all multivariable models: demographic characteristics (age [continuous, years] and sex [male or female]) and laboratory markers (hemoglobin [g/L], lymphocyte percentage [%], albumin [g/L], creatinine [mmol/L], D-dimer [μg/mL], and bicarbonate [HCO_3_^–^, mmol/L]). All laboratory values were standardized to international units where applicable.

### Data Collection and Quality Assurance

Data were retrospectively extracted from the hospital EMR system. Diagnoses were coded according to the *International Statistical Classification of Diseases, 10th revision* (*ICD-10*). Data quality was ensured by double independent entry, logical verification, and routine random review of 10% of all records. All data were fully anonymized and deidentified before analysis.

### Statistical Analysis

Baseline characteristics across the 3 study periods (2019, 2021, and 2023) were compared using 1-way ANOVA for normally distributed continuous variables, the Kruskal-Wallis *H* test for nonnormally distributed variables, and the chi-square test for categorical variables. Cox proportional hazards models were constructed to evaluate the association between pandemic phases and ICU admission risk, with the time scale defined as the interval from ED arrival to ICU admission. The proportional hazards assumption was validated using Schoenfeld residuals (global test *P*>.05). Results are reported as hazard ratios (HRs) with corresponding 95% CIs. Sensitivity analyses were conducted to verify the robustness of the primary findings: (1) temporal stratification of 2021 (first half vs second half) to assess within-year heterogeneity, (2) CCA using only participants with complete covariate data, and (3) E-value analysis to quantify potential unmeasured confounding for the primary comparison. All statistical tests were 2-sided, with the significance level set at α=.05. Statistical analyses were performed using R software (version 4.2; R Foundation for Statistical Computing) with the *survival*, *MICE*, and *EValue* packages.

### Ethical Considerations

This study was approved by the Ethics Committee of Henan Provincial People’s Hospital (2023-42). Informed consent was waived due to the retrospective observational design. All patient data were deidentified prior to analysis in compliance with the Health Insurance Portability and Accountability Act (HIPAA) Safe Harbor standard. Direct personal identifiers (full names, medical record numbers, admission IDs, and contact details) were permanently deleted and replaced with unique sequential study codes. The code-identifier linkage was stored on a password-protected institutional server, with access limited solely to the assigned data manager. Exact birth dates, free-text clinical notes, and identifiable images were excluded; age was retained as a continuous variable. The full deidentification process was audited by the hospital data security officer to ensure that no individual could be identified from the final dataset. No compensation was provided to participants. The manuscript contains no identifiable patient images or personal information.

### Reporting Guidelines

This study was reported in accordance with the Strengthening the Reporting of Observational Studies in Epidemiology (STROBE) statement for observational cohort studies and the American Psychological Association (APA) Journal Article Reporting Standards (JARS)–Quant for quantitative research. Completed checklists are provided in the supplementary materials.

## Results

### Participant Characteristics

**Figure 1.**
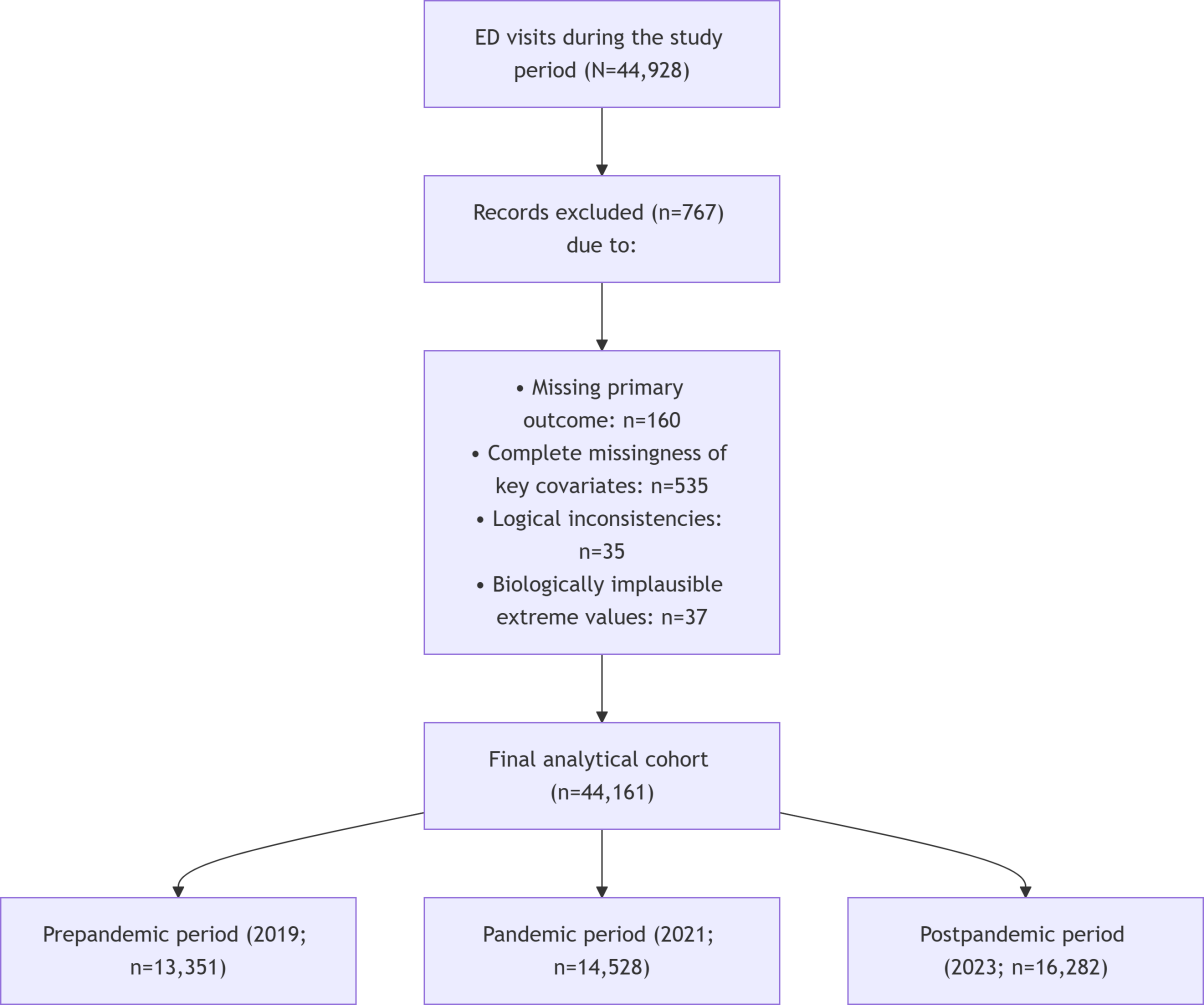
Study flow diagram. A total of 44,928 patients were initially admitted to the emergency department (ED); 767 were excluded because of a missing primary outcome, missing key covariates, logical inconsistencies, or biologically implausible extreme values. The final cohort of 44,161 patients was stratified into 3 periods: prepandemic (2019), pandemic (2021), and postpandemic (2023) periods.

A total of 44,928 ED visits were recorded during the study period; 767 invalid records were excluded, yielding a final analytical cohort of 44,161 patients (prepandemic phase: n=13,351, 30.23%; pandemic phase: n=14,528, 32.9%; and postpandemic phase: n=16,282, 36.87%; Figure 1). Baseline characteristics, laboratory parameters, and clinical outcomes across the 3 periods are summarized in Table 1.

**Table 1.** Baseline clinical and outcome characteristics of emergency department (ED) patients across COVID-19 prepandemic, pandemic, and postpandemic periods (N=44,161)^a^.

Characteristics	All	2019 (prepandemic period; n=13,351)	2021 (pandemic period; n=14,528)	2023 (postpandemic period; n=16,282)	*P* value
Age (years), mean (SD)	57.12 (20.08)	56.75 (19.89)	56.98 (19.94)	57.55 (20.35)^b,c^	.002^b,c^
Male, n (%)	24,570 (55.64)	7344 (55.01)	8082 (55.63)	9144 (56.16)^b^	<.001^b^
Hemoglobin (g/L), mean (SD)	120.1 (24)	121.7 (22.8)	120.3 (24.3)^b^	118.7 (24.6)^b,c^	<.001^b,c^
Lymphocyte leukocytes percentage (%), mean (SD)	17.98 (6.67)	19.04 (7.2)	18.14 (6.56)^b^	16.97 (6.15)^b,c^	<.001^b,c^
Albumin (g/L), mean (SD)	36.6 (6.30)	36.68 (6.24)	36.65 (5.7)	36.48 (6.84)^b,c^	.009^b,c^
Creatinine (μmol/L), mean (SD)	91.94 (95.47)	85.75 (83.98)	92.82 (95.47)^b^	97.24 (105.2)^b,c^	<.001^b,c^
D-dimer (μg/mL), mean (SD)	0.89 (1.11)	0.86 (1)	0.89 (1.18)	0.9 (1.14)^b,c^	.002^b,c^
Bicarbonate (mmol/L), mean (SD)	20.64 (3.79)	21.16 (4.06)	21 (3.22)^b^	19.9 (3.91)^b,c^	<.001^b,c^
ED length of stay (minutes), mean (SD)	387.32 (504.63)	325.56 (445.7)	390.79 (418.35)^b^	434.88 (605.18)^b,c^	<.001^b,c^
In-hospital cardiac arrest, n (%)	355 (0.8)	94 (0.7)	104 (0.72)	157 (0.96)^b,c^	.02^b,c^
Admission, n (%)	29,958 (67.83)	8719 (65.3)	10,295 (70.86)^b^	10,944 (67.22)^b,c^	<.001^b,c^
General ward admission, n (%)	22,976 (52.03)	6870 (51.46)	7778 (53.34)^b^	8328 (51.15)^c^	<.001^b,c^
Intensive care unit admission, n (%)	6982 (15.81)	1849 (13.85)	2517 (17.33)^b^	2616 (16.07)^b^	<.001^b^
Discharge, n (%)	13,800 (31.25)	4494 (33.66)	4130 (28.43)^b^	5176 (31.79)^b,c^	<.001^b,c^
Death, n (%)	167 (0.38)	42 (0.31)	53 (0.36)	72 (0.44)	.20
Transfer, n (%)	198 (0.45)	96 (0.72)	47 (0.32)^b^	55 (0.34)^b^	<.001^b^

a Continuous variables are presented as mean (SD), and categorical variables are presented as n (%). Comparisons among the 3 groups were performed using 1-way ANOVA for continuous variables and the chi-square test for categorical variables.

b *P*<.05 vs the 2019 (prepandemic phase) group.

c *P*<.05 vs the 2021 (pandemic phase) group.

Patients in the postpandemic period were significantly older than those in the prepandemic and pandemic periods (mean 57.55, SD 20.35 vs mean 56.75, SD 19.89 and mean 56.98, SD 19.94 years; *P*=.002). The proportion of male patients was also higher in the postpandemic group (9144/16,282, 56.16%) than in the prepandemic group (7344/13,351, 55.01%; *P*<.001). All laboratory indices differed significantly across groups (all *P*<.05). Hemoglobin, lymphocyte percentage, albumin, and bicarbonate (HCO_3_^–^) decreased sequentially, whereas creatinine and D-dimer increased continuously from 2019 to 2023. ED LOS increased progressively over time (mean 325.56, SD 445.70 minutes; mean 390.79, SD 418.35 minutes; and mean 434.88, SD 605.18 minutes; *P*<.001). Hospital and ICU admission rates were significantly higher in the pandemic and postpandemic periods than in the prepandemic period (all *P*<.001). ED discharge rates were lowest during the pandemic and partially recovered during the postpandemic period (*P*<.001). Interhospital transfer rates declined markedly after 2019 (*P*<.001). The incidence of IHCA was significantly higher in the postpandemic period (157/16,282, 0.96%) than in the prepandemic (94/13,351, 0.7%) and pandemic (104/14,528, 0.72%) periods (*P*=.02). In-hospital mortality showed a nonsignificant upward trend across the 3 periods (42/13,351, 0.31%; 53/14,528, 0.36%; and 72/16,282, 0.44%; *P*=.20).

### Analysis of ED Disposition Outcomes and LOS

A total of 44,123 eligible ED patients were stratified by pandemic phase and disposition: 13,351 (30.3%) in the prepandemic period, 14,525 (32.9%) during the pandemic, and 16,247 (36.8%) in the postpandemic period. Disposition groups included discharge (13,800/44,123, 31.3%), general admission (22,976/44,123, 52.1%), ICU admission (6982/44,123, 15.8%), and other dispositions (365/44,123, 0.8%).

The ED discharge rate decreased from 33.66% (4494/13,351; prepandemic phase) to 28.43% (4130/14,525; pandemic phase) and recovered to 31.86% (5176/16,247; postpandemic phase). The ICU admission rate rose from 13.85% to 17.33% during the pandemic period and then slightly declined to 16.1% during the postpandemic period. The proportion of general ward admissions remained stable across all periods.

Median ED LOS was compared using the Kruskal-Wallis *H* test with Bonferroni correction (Table 2).

**Table 2.** Emergency department (ED) disposition and median ED length of stay (LOS) across prepandemic, pandemic, and postpandemic periods (N=44,161)^a^.

ED dispositions and variables	2019 (prepandemic period; n=13,351)	2021 (pandemic period; n=14,525)	2023 (postpandemic period; n=16,247)	*P* value^b^	Pairwise comparisons^c^, *P* value		
					2019 vs 2021	2019 vs 2023	2021 vs 2023
Discharge				<.001	.047	<.001	.79
Patients, n (%)	4494 (33.66)	4130 (28.43)	5176 (31.86)				
ED LOS (minutes), median (IQR)	248 (149-404.75)	262 (153-423.75)	258 (152-451)				
General ward admission				<.001	<.001	<.001	<.001
Patients, n (%)	6870 (51.46)	7778 (53.55)	8328 (51.26)				
ED LOS (minutes), median (IQR)	186 (96-431)	350 (188-610)	293 (113-840)				
Intensive care unit admission				<.001	<.001	.003	<.001
Patients, n (%)	1849 (13.85)	2517 (17.33)	2616 (16.1)				
ED LOS (minutes), median (IQR)	95 (57-169)	171 (77-306)	105 (50-215)				
Other				.30	.38	>.99	.78
Patients, n (%)	138 (1.03)	100 (0.69)	127 (0.78)				
ED LOS (minutes), median (IQR)	220 (104-548)	182.5 (74.75-400.75)	226 (98.5-478)				

a Categorical data are presented as n (%), and continuous data are presented as median (IQR).

b Comparisons across the 3 study periods were performed using the Kruskal-Wallis *H *test.

c Post hoc pairwise comparisons were conducted using the Bonferroni correction.

In the discharge group, median ED LOS increased from 248 (IQR 149-404.8) minutes (prepandemic period) to 262 (IQR 153-423.8) minutes (pandemic period; *P*=.047) and remained elevated at 258 (IQR 152-451) minutes (postpandemic period; *P*<.001 vs the prepandemic period). In the general admission group, median ED LOS nearly doubled from 186 (IQR 96-431) minutes (prepandemic period) to 350 (IQR 188-610) minutes (pandemic period) and decreased to 293 (IQR 113-840) minutes (postpandemic period); all pairwise comparisons were significant (all *P*<.001). In the ICU admission group, median ED LOS increased from 95 (IQR 57-169) minutes (prepandemic period) to 171 (IQR 77-306) minutes (pandemic period) and returned to 105 (IQR 50-215) minutes (postpandemic period); all pairwise comparisons were significant (all *P*<.003). In the other groups, no significant interperiod difference in median ED LOS was observed (*P*=.30).

### Cox Proportional Hazards Analysis for ICU Admission

Cox proportional hazards regression was performed to evaluate ICU admission risk across pandemic phases using 3 sequentially adjusted models, with the 2019 prepandemic period as the reference (Table 3). After excluding 38 cases with missing data, 44,123 patients were included in the overall analysis (female: n=19,572, 44.36%; male: n=24,542, 55.62%).

**Table 3.** Cox proportional hazards models for the risk of intensive care unit admission across the COVID-19 pandemic phases (N=44,123).

Models and variables	All patients		Female patients (n=19,572)		Male patients (n=24,542)	
	HR^a^ (95% CI)	*P* value	HR (95% CI)	*P* value	HR (95% CI)	*P* value
Model 1 (unadjusted)						
Prepandemic phase (2019; reference)	1 (reference)	—^b^	1 (reference)	—	1 (reference)	—
Pandemic phase (2021)	1.045 (0.98‐1.115)	.18	1.128 (1.013‐1.256)	.03	0.993 (0.917‐1.076)	.86
Postpandemic phase (2023)	1.098 (1.031‐1.169)	.003	1.165 (1.048‐1.294)	.005	1.055 (0.976‐1.14)	.18
Model 2 (adjusted for age and sex)						
Prepandemic phase (2019; reference)	1 (reference)	—	1 (reference)	—	1 (reference)	—
Pandemic phase (2021)	1.035 (0.970‐1.103)	.30	1.117 (1.003‐1.244)	.04	0.991 (0.915‐1.073)	.82
Postpandemic phase (2023)	1.083 (1.017‐1.153)	.01	1.146 (1.032‐1.274)	.01	1.05 (0.971‐1.135)	.22
Male sex (vs female)	1.413 (1.341‐1.487)	<.001	—	—	—	—
Age (per 1-year increase)	1.004 (1.003‐1.005)	<.001	1.008 (1.006‐1.01)	<.001	1.002 (1‐1.003)	.03
Model 3 (fully adjusted)^c^						
Prepandemic phase (2019; reference)	1 (reference)	—	1 (reference)	—	1 (reference)	—
Pandemic phase (2021)	1.032 (0.967‐1.1)	.34	1.112 (0.999‐1.239)	.053	0.978 (0.902‐1.059)	.59
Postpandemic phase (2023)	1.079 (1.012‐1.15)	.02	1.143 (1.027‐1.273)	.02	1.028 (0.95‐1.113)	.53
Male sex (vs female)	1.413 (1.342‐1.488)	<.001	—	—	—	—
Age (per 1-year increase)	1.004 (1.003‐1.005)	<.001	1.005 (1.003‐1.007)	<.001	1.003 (1.001‐1.005)	<.001

a HR: hazard ratio.

b Not applicable.

c Fully adjusted for age, sex, hemoglobin (g/L), lymphocyte percentage (%), albumin (g/L), creatinine (mmol/L), D-dimer (μg/mL), and bicarbonate (mmol/L).

In the overall cohort, the postpandemic period (2023) was associated with an increased ICU admission risk in the unadjusted model (model 1: HR 1.098, 95% CI 1.031-1.169; *P*=.003), after adjustment for age and sex (model 2: HR 1.083, 95% CI 1.017-1.153; *P*=.01), and after full adjustment for laboratory covariates (model 3: HR 1.079, 95% CI 1.012-1.150; *P*=.02). No significant association was found for the pandemic period (2021) in any model (all *P*>.05).

In female patients, both the pandemic (2021) and postpandemic (2023) periods were linked to elevated ICU admission risk in most models. In model 1, HR values were 1.128 (95% CI 1.013‐1.256; *P*=.03) for 2021 and 1.165 (95% CI 1.048‐1.294; *P*=.005) for 2023. After full adjustment (model 3), the postpandemic period remained an independent risk factor (HR 1.143, 95% CI 1.027‐1.273; *P*=.02).

In male patients, no significant association between pandemic phase and ICU admission risk was observed in any model (all *P*>.05). Male sex (HR 1.413, 95% CI 1.342‐1.488; *P*<.001) and older age (HR 1.004, 95% CI 1.003‐1.005; *P*<.001) were independent predictors of higher ICU admission risk. None of the laboratory parameters in the fully adjusted model showed a significant association with ICU admission in male patients.

### Sensitivity Analyses

All sensitivity analyses (stratification of 2021 into 2 halves and exclusion of extreme ED LOS values) produced results consistent with the primary analysis, with the postpandemic period (2023) remaining significantly associated with ICU admission (all *P*<.05). Detailed results are presented in Table S2 in Multimedia Appendix 2.

## Discussion

### Principal Findings

The present study aimed to investigate temporal changes in ED LOS, patient disposition, and ICU admission risk among ED resuscitation patients across the prepandemic, pandemic, and postpandemic periods in central China. Three core findings were identified: ED LOS was progressively prolonged and exhibited distinct patterns across different disposition groups; the postpandemic period was independently associated with an elevated risk of ICU admission, while the pandemic period showed no significant association; and the increased ICU admission risk was predominantly driven by female patients, with no period-related changes observed in male patients. These findings highlight the sustained and heterogeneous impacts of the COVID-19 pandemic on emergency care efficiency and critical care use.

The progressive prolongation of overall ED LOS throughout the study period is consistent with global observations of pandemic-related disruptions to emergency care workflows [22-25]. Notably, the stratified analysis revealed divergent LOS trajectories across disposition groups, which have rarely been reported in previous studies. Patients admitted to general wards experienced the most severe prolongation during the pandemic, with only partial recovery in the postpandemic period. This pattern is likely attributable to downstream bed shortages and inpatient admission bottlenecks, which are known to exacerbate ED crowding and boarding [26]. In contrast, ED LOS for ICU-admitted patients increased sharply during the pandemic but nearly returned to prepandemic levels during the postpandemic period, suggesting that critical care pathways were prioritized and optimized to mitigate delays for the most severely ill patients. These divergent trends indicate that pandemic-related strain affected noncritical and critical care pathways differently, with long-lasting impacts on general ward admissions.

Prolonged ED boarding for noncritically ill patients has well-documented adverse clinical consequences, including increased mortality and worse functional outcomes [27-29]. Studies have reported that sequential capacity-building interventions—such as staff training, space reorganization, and workflow optimization—successfully reduced ED crowding and waiting times during the pandemic [30]. The marked prolongation of ED LOS for patients admitted to general wards in our study reinforces the need for targeted strategies to alleviate inpatient bed bottlenecks and streamline ED-to-ward transitions, even after the acute pandemic phase. Such interventions are critical for preventing secondary harm from delayed inpatient care in the postpandemic era.

Our analysis confirmed that the postpandemic period was associated with a significantly higher risk of ICU admission, a finding that aligns with growing evidence of persistent pandemic-related deterioration in acute care outcomes worldwide [23]. Adverse consequences of the pandemic on acute care outcomes persisted well after official containment measures were lifted [31]. This sustained elevation in critical care risk suggests that the health care system had not fully recovered to its prepandemic baseline function by 2023, likely due to lingering increases in patient acuity and delayed health care–seeking behaviors consistent with reports of persistently excess mortality in many countries long after the acute pandemic phase subsided [32,33]. Notably, no significant association was observed between the pandemic period and ICU admission risk, which may reflect the stabilizing effect of China’s dynamic zero-COVID policy in 2021, characterized by standardized NPIs and systematic patient triage, which may have attenuated the impact of pandemic-related disruptions on ICU admission risk [34]. During this period, strict containment measures may have reduced community transmission of other infectious diseases and maintained relatively stable ED care pathways for critically ill patients [2].

The most novel and clinically meaningful finding was the sex-specific disparity in ICU admission risk. Female patients exhibited persistently elevated ICU admission risk during both the pandemic and postpandemic periods, whereas male patients showed no period-related changes despite male sex being a strong independent predictor of ICU admission. This pattern suggests that women were disproportionately vulnerable to pandemic-related care disruptions, with effects persisting into the postpandemic period. Several mechanisms may explain this sex disparity. First, gendered caregiving roles may have constrained women’s health care–seeking behavior during the pandemic [35,36]. Second, implicit triage bias during periods of health care strain may have contributed. Evidence suggests that women presenting with acute cardiovascular symptoms are often evaluated less aggressively than men [37]; such biases might have been amplified during pandemic surges, leading to later recognition of critical illness in female patients. These findings highlight the need for sex-specific risk stratification and targeted interventions to identify high-risk female patients in the ED. To assess the robustness of these sex-specific findings to unmeasured confounding, we calculated E-values. For female patients, the E-values were 1.51 for 2021 vs 2019 and 1.60 for 2023 vs 2019. These values fall into the moderate range, suggesting that residual confounding cannot be entirely ruled out. Future studies with richer covariate data, including detailed psychosocial measures and sex-disaggregated triage scores, are needed to confirm whether this sex-specific vulnerability is a reproducible phenomenon.

### Limitations

Several limitations should be acknowledged. First, this single-center retrospective design limits the generalizability of the findings to other regions or health care systems with different pandemic responses. Second, residual confounding cannot be excluded, as detailed illness severity scores, ED crowding metrics, and socioeconomic factors were not available. Third, the borderline significance of pandemic period risk in female patients requires cautious interpretation and validation in future studies. Fourth, specific indications for ICU admission were not analyzed, which may limit mechanistic interpretation. Fifth, because death from non-ICU causes prior to ICU admission acts as a competing risk, the traditional Cox model might overestimate the incidence of ICU admission. Future research should use the Fine-Gray model to validate our findings, as this issue was not addressed in our analysis. Finally, the postpandemic observation period may still reflect residual pandemic effects, and longer-term follow-up is needed to assess full recovery trends.

### Conclusions

The COVID-19 pandemic exerted heterogeneous and sustained effects on ED LOS across disposition groups and increased ICU admission risk, particularly among female patients. ED workflow disruptions persisted most notably for patients who are not critically ill, while critical care pathways showed partial recovery. As a novel sex-stratified analysis covering the full pandemic timeline, this study differs from prior work by demonstrating the long-term impacts of pandemic control policies on emergency care. Our findings not only provide valuable evidence for patient risk stratification but also offer practical implications for optimizing health care resource allocation in the postpandemic era. These results support the implementation of sex-sensitive ED triage strategies, streamlined inpatient admission pathways, and targeted resource allocation for high-risk patient groups. Future multicenter prospective studies are warranted to validate these findings and clarify the mechanisms underlying sex-specific vulnerability to pandemic-related emergency care disruptions.

## Supplementary material

10.2196/87597 Multimedia Appendix 1 Cox proportional hazards regression analysis of risk factors for intensive care unit admission after multiple imputation for missing outcome data.

10.2196/87597 Multimedia Appendix 2 Sensitivity analyses for the association between pandemic phases and risk of intensive care unit admission.

## Abbreviations

APA: American Psychological Association
CCA: complete case analysis
ED: emergency department
EMR: electronic medical record
EMS: emergency medical services
HIPAA: Health Insurance Portability and Accountability Act
HR: hazard ratio
*ICD-10*: *International Statistical Classification of Diseases, 10th revision*
ICU: intensive care unit
IE: infective endocarditis
JARS: Journal Article Reporting Standards
LOS: length of stay
MAR: missing at random
MICE: multiple imputation by chained equations
NPI: nonpharmaceutical intervention
OHCA: out-of-hospital cardiac arrest
STROBE: Strengthening the Reporting of Observational Studies in Epidemiology
